# The Roots of *Deguelia nitidula* as a Natural Antibacterial Source against *Staphylococcus aureus* Strains

**DOI:** 10.3390/metabo12111083

**Published:** 2022-11-08

**Authors:** Suzana Helena Campelo Nogueira-Lima, Paulo Wender P. Gomes, Kely C. Navegantes-Lima, José Diogo E. Reis, Alice Rhelly Veloso Carvalho, Sônia das Graças Santa R. Pamplona, Abraão de Jesus B. Muribeca, Milton N. da Silva, Marta C. Monteiro, Consuelo Yumiko Yoshioka e Silva

**Affiliations:** 1 Institute of Health Sciences, Postgraduate Program in Pharmaceutical Sciences, Federal University of Pará, Belém 66075-110, Brazil; 2 Collaborative Mass Spectrometry Innovation Center, University of California San Diego, La Jolla, San Diego, CA 92093, USA; 3 Skaggs School of Pharmacy and Pharmaceutical Sciences, University of California, La Jolla, San Diego, CA 92093, USA; 4 Institute of Health Sciences, Postgraduate Program in Neuroscience and Molecular Biology, Federal University of Pará, Belém 66075-110, Brazil; 5 Institute of Exact and Natural Sciences, Postgraduate Program in Chemistry, Federal University of Pará, Belém 66075-110, Brazil; 6 Institute of Health Sciences, Faculty of Pharmaceutical Sciences, Federal University of Pará, Belém 66075-110, Brazil

**Keywords:** antibacterial activity, *Staphylococcus aureus*, amino acid derivative, liquid chromatography–mass spectrometry

## Abstract

*Deguelia nitidula* (Benth.) A.M.G.Azevedo & R.A.Camargo (Fabaceae) is an herbaceous plant distributed in the Brazilian Amazon, and it is called “raiz do sol” (sun roots). On Marajó Island, quilombola communities use its prepared roots to treat skin diseases commonly caused by fungi, viruses, and bacteria. Thus, in this study, the extract, and its fractions from *D. nitidula* roots were used to perform in vitro cytotoxic and antibacterial assays against *Staphylococcus aureus* strains. Thereafter, liquid chromatography–mass spectrometry (LC–MS) was used for the metabolite annotation process. The ethanolic extract of *D. nitidula* roots show significant bactericidal activity against *S. aureus* with IC_50_ 82 μg.mL^−1^ and a selectivity index (SI) of 21.35. Furthermore, the SREFr2 and SREFr3 fractions show a potent bactericidal activity, i.e., MIC of 46.8 μg.mL^−1^ for both, and MBC of 375 and 93.7 μg.mL^−1^, respectively. As showcased, SREFr3 shows safe and effective antibacterial activity mainly in respect to the excellent selectivity index (SI = 82.06). On the other hand, SREFr2 shows low selectivity (SI = 6.8), which characterizes it as not safe for therapeutic use. Otherwise, due to a limited amount of reference MS^2^ spectra in public libraries, up to now, it was not possible to perform a complete metabolite annotation. Despite that, our antibacterial results for SREFr3 and correlated substructures of amino acid derivatives show that the roots of *D. nitidula* are a natural source of specialized metabolites, which can be isolated in the future, and then used as a support for further bio-guided research, as well as natural drug development.

## 1. Introduction

In the last two decades, the increasing number of bacterial infections has been one of the main focuses of the World Health Organization [1]. Overall, bacterial infections are established because of the failure of the body’s immune protection mechanisms, which are the primary responses of the human organism against infections [2]. Thus, clinical microbiology studies focused on eradicating the origin of the infection, as well as on avoiding its recurrence by the administration of antibiotics that are able to inhibit the multiplication of bacteria [3]. Nevertheless, the wrong use of these drugs results in the recurrence of infections, because it enables bacterial resistance, which, in turn, compromises the effectiveness of prevention and clinical treatment, in addition to increasing hospital costs due to prolonged recovery [4].

This is why the medical sciences already consider that we live in a post-antibiotic era and that the super bacterial infections need to be treated with a level of urgency [5], since common medical procedures, such as transplants and surgeries, can become high-risk mortality practices [6]. Among these concerns, skin infections, despite being common occurrences, can progress to an irreversible clinical stage when the infectious agent is ineffective. One of the main multi-resistant bacteria, *Staphylococcus aureus*, has shown a high level of dissemination because it is usually present in the skin and mucous. However, when it is neglected, it can progress to endocarditis, meningitis, pneumonia, and central nervous system diseases [7,8]. In addition, resistant bacteria such as *S. aureus* are responsible for high morbidity and mortality in the world due to socioeconomic factors such as low-income, overcrowded and low levels of schooling, and difficulty in accessing multi-professional teams, which, in consequence, increased costs to the health system [9].

For this reason, the bioprospecting of new natural antimicrobial herbal medicines has become rather common, mainly in traditional populations, in which, due to the unavailability of basic health care, many people use medicinal plants to treat many diseases [1]. From this perspective, we believe that rational exploration from an ethnopharmacological basis is the new state-of-the-art technique for the valorization of biodiversity in a sustainable way [10]. Based on this, an ethnopharmacological study was conducted by our group in the remaining quilombola communities, where the roots of the species *D. nitidula* (Benth.) A.M.G. Azevedo & R.A. Camargo were reported among some plants cited to treat skin diseases [11]. This species is popularly known as “raiz do sol”, “sun root”, and the name was given because, according to the interviewees, its roots submerge deeply after the twelfth hour of the day. It is also recommended to collect it in the morning, at the risk of not observing the desired effect. For this use, according to local reports, the roots are washed and scraped. These scraps are dipped in alcohol and left to infuse for a few days. After this, the infusion is applied, with the help of absorbent cotton, directly to the affected skin. Furthermore, anti-inflammatory, insecticidal, and antimicrobial activities [12] have been attributed to the genus, and activities against the tick *Rhipicephalus* (*Boophilus*) *microplus* (Canestrini, 1887) [13] and acaricides [14] were reported for that species.

Complementarily, the genus *Deguelia* is mainly known for the biosynthesis of flavones and isoflavones, which are promising classes of compounds in the race for the discovery and development of new antimicrobial agents [15,16,17]. However, a comprehensive and reliable screening of complex matrices is still a challenging task, and one reason for this is that from isolation to structure elucidation much time is needed, i.e., using techniques such as chromatography in the column, high-performance liquid chromatography (HPLC), and nuclear magnetic resonance (NMR). Although NMR remains the unique technique for the complete elucidation of a chemical structure, prominent new approaches were launched in the last two decades, e.g., Global Natural Product Social Molecular Networking (GNPS) platform [18,19], which allows searching for reference MS/MS spectra, and based on this, currently, scientific reports have reported a comprehensive chemical screening of natural products from complex samples by liquid chromatography coupled to high-resolution mass spectrometry (LC–MS), associated with bioinformatics approaches [20].

Hence, bioactive extracts and fractions can have their molecular profile revealed in a few weeks or months by metabolite annotation (levels 2 and 3) [21], as established in previous reports by our research group [22,23,24]. In this approach here, we describe the chemical composition and in vitro antibacterial activity against *S. aureus* for the ethanolic extract and its fractions from the roots of *D. nitidula*.

## 2. Materials and Methods

### 2.1. Collect, Botanical Identification and Extraction

The roots of *D. nitidula* were collected in a remaining quilombola community located in the municipality of Salvaterra, Marajó, Pará (0°45′21″ S 48°45′54″ W) in January 2019. Identification of the botanical material was performed at the Empresa Brasileira de Pesquisa Agropecuária da Amazônia Oriental (EMBRAPA) and an exsiccate was deposited at the Herbarium IAN (voucher 199349). The authorization to access the traditional knowledge was registered in SISGEN (ID SISGEN A8CC64B). The roots were dried in an oven at 45 °C followed by the grinding process, where 1.0 kg of dried material was extracted with 4 L of 99.9% (*v*/*v*) ethanol. After extraction, 398 g were recovered (39.8% yield). A total of 320 g of ethanolic extract (SREE) was then fractionated using chromatography in column and silica gel as stationary phase. For this, a glass column (internal diameter = 2.5; high = 4.0) filled with the G60 silica gel adsorbent containing particles ranging from 60–200 µm (70–230 mesh) purchased from Siliciclo (Quebec City, QC, Canada) in a ratio of 1:10 (sample/silica) was used. During fractionation process, four fractions were collected using hexane, dichloromethane, ethyl acetate and methanol as mobile phase. All solvents HPLC/Spectro grade were purchase from Tedia Company (Fairfield, OH, USA). Thereafter, the organic solvents were then removed using a rotative evaporator Buchi (Valinhos, São Paulo, Brazil). Lastly, four resulting fractions were named SREFr1 (70.5 g), SREFr2 (20.28 g), SREFr3 (60.54 g), and SREFr4 (20.94 g), respectively.

### 2.2. Analysis by Liquid Chromatography–Mass Spectrometry LC–MS/MS

Liquid chromatography–mass spectrometry (LC–MS/MS) analyses were performed using liquid chromatography coupled to a Xevo G2-S Q-Tof mass spectrometer (Waters Corp., Milford, MA, USA) equipped with an electrospray source. The samples were analyzed in a BEH C18 column (Waters Corp.; 50 mm; 2.1 mm; 1.7 μm particle size) using ultra-pure water (solvent A) and acetonitrile (solvent B) as mobile phases, both acidified with 0.1% formic acid. The column temperature was set at 40 °C and a flow rate of 0.3 mL/min. A total of 2 μL of extract (1 mg.mL^−1^) was analyzed in the UPLC system, using gradient elution from 5% to 95% solvent B, for 11 min. Leucine-enkephalin (Waters Corp., Milford, MA, USA) was used as a reference mass. The source temperature was set up to 120 °C with a cone gas flow of 30 L/h. The desolvation gas flow was set up to 600 L/h at a temperature of 250 °C. The capillary sampling was set up to 3.5 kV with the cone voltage of 40 V. A DDA method data was used in positive mode (top5 most intense ions), mass range from *m/z* 50 to 1200 Da (MS^1^ and MS^2^) with a scan time of 0.1 s. A profile of normalized collision energy (10–40 eV) was applied and MassLynx software (Waters Corp., Milford, MA, USA) was used to control the system and data acquisition.

### 2.3. Data Analysis

A processed quantification table (CSV) and MGF files from MZmine 3.0 [25] containing 850 features were then used to search for reference MS/MS spectra in GNPS libraries [18], as well as to carry out statistical analysis in Jupyter notebook. Additionally, Sirius 5.0 [26] was used to predict the substructures.

### 2.4. Ethics Statement

For in vitro toxicity test, human venous blood was collected from healthy volunteers who were abstainers of alcohol and tobacco (both sexes, ages 20 to 45 years) that signed the informed consent form (ICF). This study was approved by the Institutional Committee of Ethics in Research involving human beings from the health sciences sector of UFPA (CEP-ICS/UFPA, under code 3.544.380). For the ethnopharmacological study, five remaining quilombola communities belonging to the city of Salvaterra, Marajó, Pará, were interviewed and this study was registered at CEPS/ICS/UFPA sector, under code CAAE: 22886619.5.0000.0018.

### 2.5. Peripheral Blood Mononuclear Cells (PBMC) Isolation

PBMC were isolated from whole blood using Histopaque-1077 (Sigma-Aldrich, St. Louis, MO, USA). Thereafter, the whole blood sample was diluted to 1:1 volume ratio with saline solution (0.9%) and centrifugated for 20 min (2000RPM). Posteriorly, PBMC viability was determined by trypan blue exclusion and the viability was always >95%. Then, the cells were washed and suspended in Roswell Park Memorial Institute-1640 medium (RPMI-1640, Sigma-Aldrich), and then it was supplemented with sodium bicarbonate (2 g.L^−1^), 10% (*v*/*v*) fetal bovine serum (FBS, Sigma-Aldrich), 2% (*v*/*v*) glutamine (Sigma-Aldrich), and, lastly, the resultant solution was incubated with RPMI 1640 Complete Medium supplemented to evaluate cell viability.

### 2.6. Cell Viability—MTT Assay

To evaluate the cytotoxicity of the ethanolic extract and its fractions from *D. nitidula* roots ethanolic extract (SREE), the cell viability of peripheral PBMC were performed by 3-(4,5-dimethylthiazol-2yl)-2,5-bromide-diphenyl-tetrazolium (MTT) assays [27]. Hence, PBMC were seeded in 96-well polystyrene culture plates at a concentration of 2 × 10^5^ mL^−1^ cells concentration using the RPMI 1640 Complete Medium supplemented and incubated with 100 μL of control (RPMI), solutions, extract, and fractions at concentration levels of 1500, 750, 375, 187.5, and 93.75 μg.mL^−1^, negative control used untreated cells and positive control used 1% Triton-X-100 (*v*/*v*). The plate was incubated for 24 h at 37 °C with 5% CO_2_, and then the medium was withdrawn to add 100 μL of the MTT solution (0.5 mg.mL^−1^) in all microtitration wells. Thereafter, the MTT solution was withdrawn and 100 μL of HCl (6 N) with isopropanol was added to all wells, and the plate reading was read and performed at an absorbance of 550 nm. For the cytotoxic concentration (CC_50_) determination, a linear regression curve was used, and the value was expressed based on absorbance in the Graphpad Prism 8 software (GraphPad Software Inc., La Jolla, San Diego, CA, USA).

### 2.7. Antibacterial Bioassay

Antibacterial properties were evaluated against Gram-positive strains of *Staphylococcus aureus* (ATCC 6538). It was obtained from INCQS/FIOCRUZ (National Institute for Quality Control in Health, Brazil). Strains were previously seeded in Petri dishes containing Mueller–Hinton agar (Merck, Darmstadt, Germany) and incubated at 37 °C for 24 h. For the preparation of the bacterial inoculum, the strains growing were performed in Mueller–Hinton (MHB) broth (Merck, Darmstadt, Germany) at 37 °C for 24 h and adjusted by dilution of fresh cultures to a turbidity equivalent to 0.5 scales of McFarland (around 2 × 10^8^ CFU.mL^−1^). and then diluted to 1 × 10^3^ CFU.mL^−1^ as described in the literature [28] and according to the Clinical and Laboratory Standard Institute [29]. Minimum inhibitory concentration (MIC) and minimum bactericidal concentration (MBC) assays were performed by the broth microdilution method in the MHB (National Committee for Clinical Laboratory Standards) [29]. The MIC was defined as the lowest concentration of the extract without visible bacterial growth in the resazurin colorimetric assay [30].

To determine the MIC, extracts were dissolved in DMSO 10% (*v*/*v*) and distilled water at the highest concentration of 1500 µg.mL^−1^ to the lowest of 46.8 µg.mL^−1^ by serial dilution. A total of 100 μL of the extract and fractions were placed in each microtitration well together with 100 μL of the bacterial inoculum (1 × 10^3^ CFU.mL^−1^). After incubation, the development of a pinkish-purple color was considered indicative of bacterial growth. Therefore, the MIC reading was set up to the lowest concentration of the extract where the pink–purple color was not observed. To determine the CBM, 10 µL of broth was taken from each well and incubated in Mueller–Hinton agar at 37 °C for 24 h for each bacterium. MBC was defined as the lowest concentration of extract that resulted in a colony count of fewer than three colonies per mL (90.0% kill) or no bacterial growth [31]. The assays were performed in triplicates. The negative control consisted of 100 μL of bacterial inoculum and 100 μL of DMSO. Chloramphenicol (50 μg.mL^−1^) was used as a positive control for Gram-positive bacteria.

To evaluate 50% inhibition concentration (IC_50_), a literature-adapted method [32] was applied by use of sterile 96-well polystyrene culture plates (Corning Costar Corp., Acton, MA, USA) with flat-bottom wells. To perform these assays, ethanolic extract and its fractions from the roots of *D. nitidula* at concentration levels of 1500, 750, 375, 187.5, 93.75, and 46.8 μg.mL^−1^ were transferred to separate microplate wells (100 μL/well) with 100 µL aliquot of *S. aureus* inoculum (1 × 10^3^ CFU.mL^−1^). As a negative control, Mueller–Hinton broth and 50 µg.mL^−1^ of chloramphenicol were used against *S. aureus* and incubated for 24 h at 37 °C. After 24 h of incubation, the absorbance was measured in an ELISA reader (BioTek Synergy HTX Multimode Reader) at 600 nm with gentle agitation for 10 s. The IC_50_ was calculated using the linear relation between the MIC and concentration logarithm. The selectivity index (SI) was calculated with the ratio of IC_50_ (against *S. aureus*) and CC_50_ (PBMC).

## 3. Results

### 3.1. In Vitro Toxicity Test

**Figure 1:**
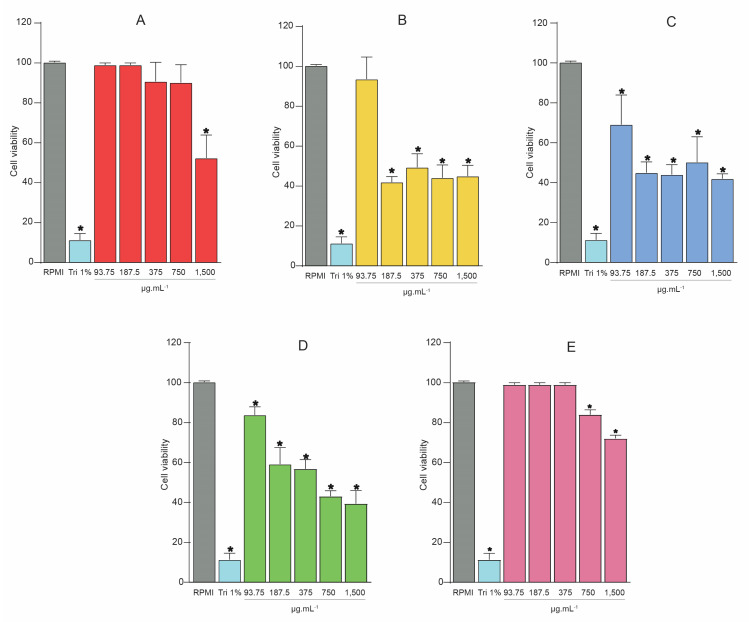
Cell viability was assessed by MTT assays with the extract and its fractions from *D. nitidula* roots. (**A**) Ethanolic extract of *D. nitidula* roots (SREE). (**B**) Hexane fraction (SREFr1). (**C**) Dichloromethane fraction (SREFr2). (**D**) Ethyl acetate fraction (SREFr3). (**E**) Methanol fraction (SREFr4). Data presented as mean ± SD. (* *p* < 0.05 extract, its fractions from *D. nitidula* roots or Triton 1% vs. RPMI control). RPMI: Gibco Roswell Park Memorial Institute 1640 Medium; Tri 1%: Triton X-100 1% (*v*/*v*).

In the present study, toxicity assays at different concentrations (93.75, 187.5, 375, 750, and 1500 μg.mL^−1^) were performed for SREE and its fractions (SREFr1, SREFr2, SREFr3, SREFr4). Thus, PBMC were used, and the CC_50_ was defined as how efficiently the extract, or its fractions could reduce 50% of the cell viability. The SREE shows a high cytotoxicity level (1500 μg.mL^−1^: 48.66 ± 11.57%) with a CC_50_ of 1751.12 μg.mL^−1^, unlike other concentrations that show cell viability. SREFr1 shows a cytotoxic profile (187.5 μg.mL^−1^ = 41.98 ± 2.78%; 375 μg.mL^−1^ = 49.37 ± 6.82%; 750 μg.mL^−1^ = 44.18 ± 6.39%, and 1500 μg.mL^−1^ = 45.01 ± 5.36%) with a CC_50_ of 571.6 μg.mL^−1^ (Figure 1B), except for the lowest level where the cell viability is maintained (93.75 μg.mL^−1^ = 93.59 ± 11.09%). Similar results are observed for SREFr3, i.e., the lowest concentration (93.75 μg.mL^−1^) shows lower cytotoxicity, and excitedly high cell viability (83.84 ± 4.12%) (see Figure 1D) with CC_50_ of 902.76 μg.mL^−1^. SREFr2 (Figure 1C) reduces cell viability at all concentrations tested, presenting a CC_50_ of 863.05 μg.mL^−1^. Otherwise, SREFr4 shows 100% viability at the lowest concentrations tested, and shows low cytotoxicity at the highest concentrations when compared to the other fractions. In addition, this fraction shows high CC_50_ 10,089.90 μg.mL^−1^ but no bacterial properties against *S. aureus* strains. In this case, our group will develop new antimicrobial studies in the future.

### 3.2. In Vitro Antibacterial Properties

The ethanolic extract of *D. nitidula* roots (SREE) and hexane fraction (SREFr1), dichloromethane fraction (SREFr2), ethyl acetate fraction (SREFr3), and methanol fraction (SREFr4) were evaluated at different concentrations against *S. aureus* to determine the MIC, MBC, and the MBC/MIC ratio in order to determine whether the antimicrobial activity have an bactericidal and bacteriostatic effect.

Table 1 shows that it was only possible to determine the MIC and MBC of SREE, SREFr2, and SREFr3, while in the other fractions, it was not possible to find MIC (SREFr4) and MBC (SREFr1 e SREFr4) values within the tested concentrations. Thus, from the obtained MIC and MBC results described in Table 1, we determined the MBC/MIC ratio. According to the literature [33], MBC/MIC ratio ≤4 presents bactericidal activity, whereas MBC/MIC ratio >4 presents bacteriostatic activity. In this context, SREE and SREFr3 show bactericidal activity and SREFr1 and SREFr2 show bacteriostatic activity. Posteriorly, the average IC_50_ of the extract and its fractions against *S. aureus* were evaluated. SREFr3 shows the lowest IC_50_ (11 μg.mL^−1^), which is able to inhibit 50% of bacterial growth, while SREFr2 shows the highest IC_50_ (128 μg.mL^−1^), as shown in Table 1.

To determine the cytotoxic and antibacterial window of the extract and its fractions, the ratio between CC_50_ and IC_50_ was calculated (Table 1). The SI of SREE (SI = 21.35) and SREFr3 (SI = 82.06) are excellent (SI ≥ 10), and they can be considered as safe and effective antibacterial. On the other hand, SREFr2 (SI = 6.8) presents low selectivity (SI ≤ 10) [34].

### 3.3. Bioactive Molecular Profile

**Figure 2:**
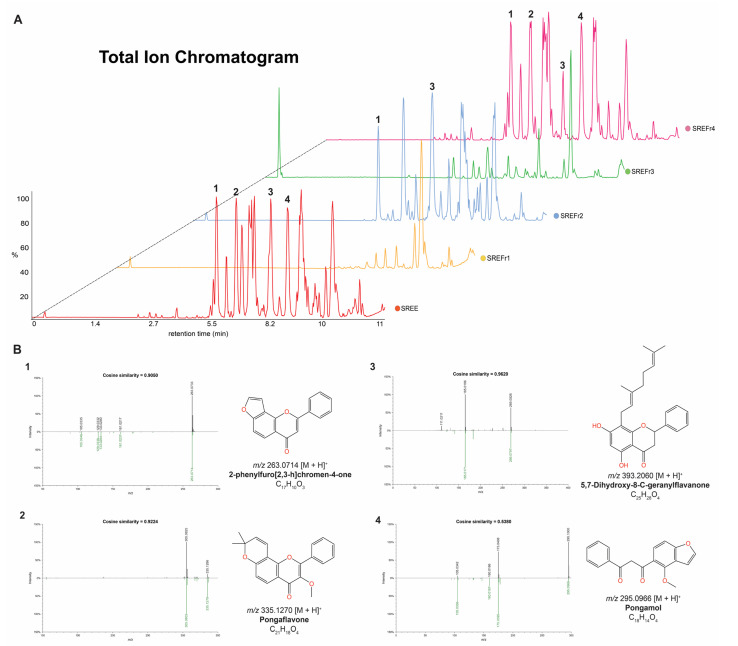
(**A**) The LC–MS fingerprints of the extract and its fractions from *D. nitidula* roots. (**B**) Matches between experimental MS/MS spectra and reference MS/MS spectra from GNPS libraries for the annotated flavonoid derivatives (1–4) in the SREE.B1-B4 in the SREE, SREFr2, and SREFr4 (link to see the mirror plots: https://gnps.ucsd.edu/ProteoSAFe/result.jsp?task=7cfcdd2029174559a7c87182189fc568&view=view_all_annotations_DB (accessed on 7 October 2022)).

Overall, the main metabolites detected in the ethanolic extract (SREE), fractions SREFr2, were putatively annotated as flavonoid derivatives, and they are illustrated in Figure 2A. Thus, modified cosine [35] was applied to assess the MS/MS correspondence between experimental spectra and reference spectra available in GNPS libraries [18]. For instance, 5,7-Dihydroxy-8-*C*-geranylflavanone (Figure 2B3) showed a fragment of *m/z* 165 that could be generated by heterocyclic ring fission (HRF) [36] in the C-ring of the precursor ion, and pongaflavone (Figure 2B2) showed loss of CH_2_O (30 Da) from the pyrano. Excitedly, suitable similarities using modified cosine >0.50 were observed for those metabolites.

**Figure 3:**
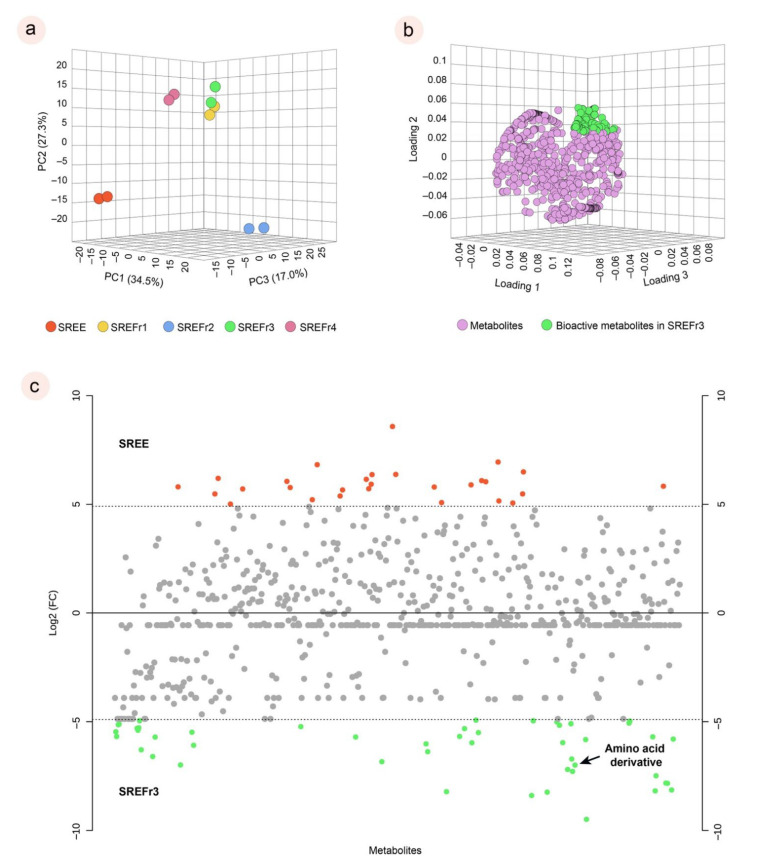
Principal component of analysis (PCA) for the extract and its fractions in duplicates (**a**); main bioactive metabolites for the SREFr3 highlighted in green (**b**); and fold change chart between SREE and SREFr3. Red points refer to metabolites in high relative concentration in SREE with at least a double change; Green points refer to metabolites in high relative concentration in SREFr3 with at least a double change; Grey points no significant fold changes were observed (**c**).

To obtain a general overview of the molecular profile from *D. nitidula* roots, we carried out a PCA. Thus, processed LC–MS data containing features with the mass-to-charge ratio (*m/z*) and retention time were used to obtain relative quantification information of the extract and its fractions. From the results of this PCA, the extract (SREE) tends to have a significant separation from its fractions, as shown in Figure 3a, and, among the fractions, notably SREFr2 can be considered as an outline. On the other hand, SREFr1, SREFr3, and SREFr4 show consistent clustering, especially between SREFr1 and SREFr3, and a group of metabolites (green highlight, Figure 3b) likely is present in both samples, at different levels of concentration.

**Figure 4:**
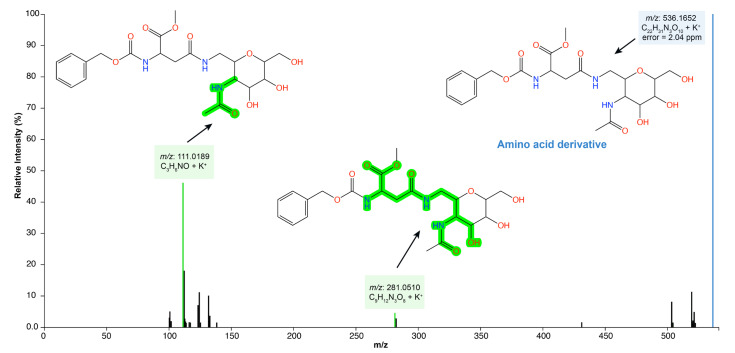
SREFr3 MS/MS spectrum highlighting annotated product ions of *m/z* 281 and *m/z* 111, which have amino acid derivatives from asparagine (green shadow).

In addition, log 2 (fold change) was performed to understand the absolute value of change in the bioactive profile between the extract and SREFr3 fraction (Figure 3c). Hence, metabolites in red color are detected in high relative concentration in the ethanolic extract (SREE) and the green highlighted metabolites from the ethyl acetate fraction (SREFr3). Among the group of metabolites related to SREFr3, in silico prediction shows evidence of the presence of substructures from amino acid derivatives (green shadow), as illustrated for the product ions of *m/z* 281 and *m/z* 111 (Figure 4).

## 4. Discussion

Herein, an in vitro screening of the extract and its fractions was performed to assess the cytotoxicity. For this, PBMC assays were performed using different concentrations (93.75, 187.5, 375, 750, and 1500 μg.mL^−1^) and the antimicrobial activity against *S. aureus,* a Gram-positive bacteria. According to a previous report [37], a plant extract has high antimicrobial activity if the MIC < 100 μg.mL^−1^; otherwise, if the values are between ≥100 μg.mL^−1^ and ≤2048 μg.mL^−1^, the extract can have moderate activity, and lastly, MIC > 2048 μg.mL^−1^ weak activities, while MIC > 10,000 μg.mL^−1^ is attributed as no antimicrobial action. The same cutoff point was determined. The SI allows measuring observed previously by [38], thus, SREE, SREFr2, and SREFr3 show strong activities against *S. aureus* (MIC = 93.7; 46.8; and 46.8 μg.mL^−1^, respectively), and the SREFr1 and SREFr4 fractions show moderate antibacterial activity (MIC = 187.50 and >1500, respectively).

To determine the cytotoxic concentration in relation to the effective bioactive concentration of a single sample, the SI was calculated. Thus, it is considered a predictive methodology for the sensitivity and specificity of the sample evaluated [39]. In this context, a safe drug must have low effective concentration and high cytotoxic concentration. Hence, our results demonstrate that the SREE has a CC_50_ of 1751.12 μg.mL^−1^ and an IC_50_ of 82 μg.mL^−1^, which shows it is safe, and an SI of 21.35 μg.mL^−1^ for *S. aureus*, which characterizes it as an effective antimicrobial when the index SI ≥ 10 [34].

The antimicrobial activity of SREE can be justified by the presence of antimicrobial components such as the flavonoid derivatives described in Figure 2. The flavonoids have anti-bacterial activity with multiple cellular targets, such as the inhibition of ATPase activity in Gram-negative bacteria through the DNA of gyraseB (GyrB) [40], and these compounds are involved in bacterial efflux pump inhibitors in Gram-positive bacteria (e.g., *S.aureus*) [41]. That confers a bactericidal effect (MBC/MIC = 2) on SREE and SREFr3.

This mechanism is important because Gram-positive bacteria such as *S. aureus* can develop resistance to antibiotics through the expression of efflux pumps to pump out antibiotics from the bacteria, for example [42]. In this regard, the SREE can improve the susceptibility of Gram-positive bacterial strains. However, some factors may contribute to the effectiveness of these compounds, especially lipophilicity, such as those found in this study, for example, pongaflavone (Figure 2B) [43,44].

Although SREFr2 shows high antibacterial activity similar to SREFr3, the IC50 of SREFr2 is significantly higher (128 μg.mL^−1^) when compared to SREFr3 (11 μg.mL^−1^). With that, this fraction shows a low selectivity index (SI = 6.8), which characterizes SREFr2 as not safe for therapeutic use. On the other hand, the SREFr3 fraction stood out in all assays, as it proved to be safe against human cells and with high antimicrobial activity, with CC_50_ values of 902.76 and MIC of 46.8 μg.mL^−1^, thus, obtaining a high value of SI against *S. aureus* (SI = 82.06), showing a promising fraction for future studies.

The molecular profile is indicative of amino acid derivatives from asparagine, which are part of the nonspecific defense system of plants, and are reported to have antimicrobial activity [45]. The antibacterial activity of this class of specialized metabolites involves a broad spectrum of bacteria including *S. aureus* [46]. Furthermore, the interaction of these metabolites with plasma membrane lipids is usually facilitated by their amphipathic structure and a positive charge in physiological pH, where their cationic residues electrostatically attract negatively charged molecules (e.g., anionic phospholipids, lipopolysaccharides, or teichoic acids), enabling the accumulation of these amino acid derivatives on the membrane surface [38]. As for the antibacterial mechanism of the action of amino acid derivatives, they usually participate in the bacterial cell wall, specifically on target enzymes involved in peptidoglycan (murein) biosynthesis, and promote the catalysis of key steps in oligopeptide production, the so-called MurA–F pathway [45], and, thus, cause interruption of the respiratory process and, consequently, cell death.

## 5. Conclusions

This study shows the ethanolic extract and ethyl acetate fraction from *D. nitidula* roots as antibacterial agents against *S. aureus* strains. Flavonoids, including pongaflavone and pongamol, were mainly described in the extract (SREE), which shows interesting antibacterial properties. Excitedly, ethyl acetate fraction (SREFr3) shows potent antibacterial properties, despite it not being possible to define putative precursor metabolites from this fraction. This activity could be explained by the in silico indicative presence of amino acid derivatives from asparagine. In addition, this chemical class, which is reported for the first time in this species, is well-known for its antibacterial properties. On the other hand, it was not possible to calculate the SI of SREFr1, SREFr2 showed significant toxicity, and SREFr4 had no activity against *S. aureus*. Hence, we show that the roots are a renewable antibacterial source, and SREE and SREFr3 can serve as a pillar for future antimicrobial studies.

## Figures and Tables

**Table 1:** Evaluation of the antibacterial activity against *S. aureus* and cytotoxicity (CC_50_), inhibition (IC_50_), and selective index (SI) of the extract and fractions of the roots of *D. nitidula*.

Extract	Code	*Staphylococcus aureus*				PBMC	
		^1^ MIC (μg.mL^−1^)	^2^ MBC (μg.mL^−1^)	MBC/MIC (μg.mL^−1^)	^3^ IC_50_ (μg.mL^−1^)	^4^ CC_50_ (μg.mL^−1^)	^5^ SI
Ethanol extract	SREE	93.7	187.0	2	94.82	1751.12	18.6221.35
Hexane fraction	SREFr1	187.050	>1500	>8.01	ND *	571.6	ND *
Dichloromethane fraction	SREFr2	46.8	375.0	8.01	180.128	863.05	4.796.8
Ethyl acetate fraction	SREFr3	46.8	93.7	2	52.11	902.76	17.3682.06
Methanol fraction	SREFr4	>1500	>1500	ND *	ND *	10,089.9	ND *
Chloramphenicol		50	ND *	ND *	25	ND *	ND *

^1^ MIC: minimum inhibitory concentration; ^2^ MBC: maximal inhibitory concentration; ^3^ IC_50_: half-maximal inhibitory concentration; ^4^ CC_50_: half maximal cytotoxic concentration; ^5^ SI: selectivity index; * ND: Not determined.

## Data Availability

The data presented in this study are available in the main article.

## References

[1] (2004). WHO Guidelines on Safety Monitoring of Herbal Medicines in Pharmacovigilance Systems.

[2] Brandão S.C.S., Godoi E.T.A.M., de Ramos J.O.X., de Melo L.M.M.P., Sarinho E.S.C. (2020). Severe COVID-19: Understanding the role of immunity, endothelium, and coagulation in clinical practice. J. Vasc. Bras..

[3] Roca I., Akova M., Baquero F., Carlet J., Cavaleri M., Coenen S., Cohen J., Findlay D., Gyssens I., Heure O.E. (2015). The global threat of antimicrobial resistance: Science for intervention. New Microbes New Infect..

[4] Foster T.J. (2017). Antibiotic resistance in *Staphylococcus aureus*. Current status and future prospects. FEMS Microbiol. Rev..

[5] Klein E.Y., Mojica N., Jiang W., Cosgrove S.E., Septimus E., Morgan D.J., Laxminarayan R. (2017). Trends in methicillin-resistant *Staphylococcus aureus* hospitalizations in the United States, 2010–2014. Clin. Infect. Dis..

[6] de Santos N.Q. (2004). A resistência bacteriana no contexto da infecção hospitalar. Texto Contexto Enferm..

[7] Taylor T.A., Unakal C.G. (2022). Staphylococcus Aureus. Stat Pearls.

[8] Ondusko D.S., Nolt D. (2018). Staphylococcus Aureus. Pediatr. Rev..

[9] Allel K., García P., Labarca J., Munita J.M., Rendic M., de Resistencia Bacteriana G.C., Undurraga E.A. (2020). So-cioeconomic factors associated with antimicrobial resistance of *Seudomonas aeruginosa*, *Staphylococcus aureus*, and *Escherichia coli* in chilean hospitals (2008–2017). Rev. Pan Am. Salud Publica.

[10] da Silva A.S.L., de Carvalho M.L.S., de Jesus Benevides C.M. (2022). Ethnopharmacological studies in 21st century Brazil: A systematic review. RSD.

[11] Silva S.H.C.N. (2020). Avaliação de atividade antimicrobiana E perfil fitoquímico de plantas medicinais utilizadas por comunidades remanescentes de quilombolas no Marajó. Mestre em Ciências Farmacêuticas.

[12] Lima N.M., Cursino-Hron L.M., Lima A.M., Souza J.V.B., de Oliveira A.C., Marinho J.V., Nunez C.V. (2018). Antifungal activity of extracts and phenolic compounds from *Deguelia duckeana*. Rev. Bras. Farmacogn..

[13] Machado A.F., Castro e Silva A., Ribeiro H.C.T., Procópio A.R.D.L., Pinheiro C.C.D.S., Martins J.R.D.S., Silva W.C. (2013). Atividade biológica de extratos acetato de etila, etanólico e aquoso de timbó (*Lonchocarpus floribundus*) sobre carrapato bo-vino. Acta Amazon.

[14] Amaral A.C.F., Ramos A.D.S., Pena M.R., Ferreira J.L.P., Menezes J.M.S., Vasconcelos G.J., da Silva N.M., Silva J.R.D.A. (2017). Acaricidal activity of derris floribunda essential oil and its main constituent. Asian Pac. J. Trop. Biomed..

[15] Shamsudin N.F., Ahmed Q.U., Mahmood S., Shah S.A.A., Khatib A., Mukhtar S., Alsharif M.A., Parveen H., Zakaria Z.A. (2022). Antibacterial effects of flavonoids and their structure-activity relationship study: A comparative interpretation. Molecules.

[16] Oliveira D.G.D., Almeida C., Silva C.Y., Arruda M.S., Arruda A.C., Lopes D.C., Yamada E.S., Costa E.T., Silva M.N. (2012). Flavonoids from the leaves of deguelia utilis (Leguminosae): *Structural elucidation* and neuroprotective properties. J. Braz. Chem. Soc..

[17] Da Costa D., Silva C.E., Pinheiro A., Frommenwiler D., Arruda M., Guilhon G., Alves C., Arruda A., Da Silva M. (2016). Using LC and hierarchical cluster analysis as tools to distinguish timbó collections into two deguelia species: A contribution to chemotaxonomy. Molecules.

[18] Wang M., Carver J.J., Phelan V.V., Sanchez L.M., Garg N., Peng Y., Nguyen D.D., Watrous J., Kapono C.A., Luzzatto-Knaan T. (2016). Sharing and community curation of mass spectrometry data with global natural products social molecular networking. Nat. Biotechnol..

[19] Aron A.T., Gentry E.C., McPhail K.L., Nothias L.-F., Nothias-Esposito M., Bouslimani A., Petras D., Gauglitz J.M., Sikora N., Vargas F. (2020). Reproducible molecular networking of untargeted mass spectrometry data using GNPS. Nat. Protoc..

[20] Bittremieux W., Avalon N.E., Thomas S.P., Kakhkhorov S.A., Aksenov A.A., Gomes P.W.P., Aceves C.M., Rodríguez A.M.C., Gauglitz J.M., Gerwick W.H. (2022). Open access repository-scale propagated nearest neighbor suspect spectral library for untargeted metabolomics. bioRxiv.

[21] Sumner L.W., Amberg A., Barrett D., Beale M.H., Beger R., Daykin C.A., Fan T.W.-M., Fiehn O., Goodacre R., Griffin J.L. (2007). Proposed minimum reporting standards for chemical analysis chemical analysis working group (CAWG) metabolomics standards initiative (MSI). Metabolomics.

[22] Gomes P.W.P., Barretto H., Reis J.D.E., Muribeca A., Veloso A., Albuquerque C., Teixeira A., Braamcamp W., Pamplona S., Silva C. (2022). Chemical composition of leaves, stem, and roots of *Peperomia pellucida* L. kunth. Molecules.

[23] Santiago J.C.C., Albuquerque C.A.B., Muribeca A.d.J.B., Sá P.R.C., Pamplona S.d.G.S.R., e Silva C.Y.Y., Ribera P.C., Fontes-Júnior E.D.A., da Silva M.N. (2022). *Margaritaria nobilis* L.F. (Phyllanthaceae): Ethnopharmacology and application of computational tools in the annotation of bioactive molecules. Metabolites.

[24] de Muribeca A.J.B., Gomes P.W.P., Paes S.S., da Costa A.P.A., Gomes P.W.P., de Viana J.S., Reis J.D.E., das Pamplona S.G.S.R., Silva C., Bauermeister A. (2022). Antibacterial activity from *Momordica charantia* L. leaves and flavones enriched phase. Pharmaceutics.

[25] Pluskal T., Castillo S., Villar-Briones A., Orešič M. (2010). MZmine 2: Modular framework for processing, visualizing, and analyzing mass spectrometry-based molecular profile data. BMC Bioinform..

[26] Dührkop K., Fleischauer M., Ludwig M., Aksenov A.A., Melnik A.V., Meusel M., Dorrestein P.C., Rousu J., Böcker S. (2019). SIRIUS 4: A rapid tool for turning tandem mass spectra into metabolite structure information. Nat. Methods.

[27] Mosmann T. (1983). Rapid colorimetric assay for cellular growth and survival: Application to proliferation and cytotoxicity assays. J. Immunol. Methods.

[28] Lopes T.R.M., de Oliveira F.R., Malheiros F.F., de Andrade M.A., Monteiro M.C., Gonçalves A.C.B. (2014). Antimicrobial bioassay-guided fractionation of a methanol extract of *Eupatorium triplinerve*. Pharm. Biol..

[29] M07: Dilution AST for Aerobically Grown Bacteria—CLSI. https://clsi.org/standards/products/microbiology/documents/m07/.

[30] Monteiro M.C., de la Cruz M., Cantizani J., Moreno C., Tormo J.R., Mellado E., de Lucas J.R., Asensio F., Valiante V., Brakhage A.A. (2012). A new approach to drug discovery: High-throughput screening of microbial natural extracts against aspergillus fumigatus using resazurin. J. Biomol. Screen..

[31] de Quadros A.U., Bini D., Pereira P.A.T., Moroni E.G., Monteiro M.C. (2011). Antifungal activity of some cyclooxygenase inhibitors on *Candida albicans*: PGE2-dependent mechanism. Folia Microbiol..

[32] Sultanbawa Y., Cusack A., Currie M., Davis C. (2009). An innovative microplate assay to facilitate the detection of antimicrobial activity in plant extracts. J. Rapid Methods Autom. Microbiol..

[33] Mogana R., Adhikari A., Tzar M.N., Ramliza R., Wiart C. (2020). Antibacterial activities of the extracts, fractions and isolated compounds from *Canarium patentinervium* Miq. against bacterial clinical isolates. BMC Complement. Med. Ther..

[34] Awouafack M.D., McGaw L.J., Gottfried S., Mbouangouere R., Tane P., Spiteller M., Eloff J.N. (2013). Antimicrobial activity and cytotoxicity of the ethanol extract, fractions and eight compounds isolated from *Eriosema robustum* (Fabaceae). BMC Complement. Altern. Med..

[35] Bittremieux W., Schmid R., Huber F., van der Hooft J.J.J., Wang M., Dorrestein P.C. (2022). Comparison of cosine, modified cosine, and neutral loss based spectrum alignment for discovery of structurally related molecules. J. Am. Soc. Mass Spectrom..

[36] Gomes P., Quirós-Guerrero L., Silva C., Pamplona S., Boutin J.A., Eberlin M., Wolfender J.-L., Silva M. (2021). Feature-based mo-lecular network-guided dereplication of natural bioactive products from leaves of *Stryphnodendron pulcherrimum* (Willd.) hochr. Metabolites.

[37] Tagousop C.N., Tamokou J.-D., Ekom S.E., Ngnokam D., Voutquenne-Nazabadioko L. (2018). Antimicrobial activities of flavonoid glycosides from Graptophyllum grandulosum and their mechanism of antibacterial action. BMC Complement. Altern. Med..

[38] Tamokou J.D.D., Mbaveng A.T., Kuete V., Kuete V. (2017). Chapter 8—Antimicrobial activities of african medicinal spices and vegetables. Medicinal Spices and Vegetables from Africa.

[39] Cushnie T.P.T., Cushnie B., Echeverría J., Fowsantear W., Thammawat S., Dodgson J.L.A., Law S., Clow S.M. (2020). Bioprospecting for antibacterial drugs: A multidisciplinary perspective on natural product source material, bioassay selection and avoidable pitfalls. Pharm. Res..

[40] Ramachandran B., Srinivasadesikan V., Chou T.-M., Jeyakanthan J., Lee S.-L. (2020). Atomistic simulation on flavonoids derivatives as potential inhibitors of bacterial gyrase of Staphylococcus aureus. J. Biomol. Struct. Dyn..

[41] Waditzer M., Bucar F. (2021). Flavonoids as inhibitors of bacterial efflux pumps. Molecules.

[42] Sharma A., Gupta V.K., Pathania R. (2019). Efflux pump inhibitors for bacterial pathogens: From bench to bedside. Indian J. Med. Res..

[43] Mukne A.P., Viswanathan V., Phadatare A.G. (2011). Structure pre-requisites for isoflavones as effective antibacterial agents. Pharmacogn. Rev..

[44] Setzer M.S., Sharifi-Rad J., Setzer W.N. (2016). The search for herbal antibiotics: An in-silico investigation of antibacterial phytochemicals. Antibiotics.

[45] Nowak M.G., Skwarecki A.S., Milewska M.J. (2021). Amino acid based antimicrobial agents—Synthesis and properties. Chem. Med. Chem..

[46] Siebert A., Wysocka M., Krawczyk B., Cholewiński G., Rachoń J. (2018). Synthesis and antimicrobial activity of amino acid and peptide derivatives of mycophenolic acid. Eur. J. Med. Chem..

